# Supplementary material for: Improvement in kidney function in patients with chronic hepatitis B and chronic kidney disease after switching to tenofovir alafenamide fumarate: a systematic review with single arm meta-analysis
Source: J Bras Nefrol. 2026 Jan 9;48(1):e20250193. doi: 10.1590/2175-8239-JBN-2025-0193en (PMC12788822; doi:10.1590/2175-8239-JBN-2025-0193en)
Supplement: Translation

# Translation: Melhora da função renal em pacientes com hepatite B crônica e doença renal crônica após a mudança para fumarato de tenofovir alafenamida: uma revisão sistemática com meta-análise de braço único

**DOI:** 10.1590/2175-8239-JBN-2025-0193pt

**Authors:** João Galdino de Pascoa, Ramon Huntermann, Victor Machado Viana Gomes, Frederico de Sousa Marinho Mendes, João Marcelo Vallim Bertozzi, Paulo Ricardo Gessolo Lins

**Affiliations:** 1 Hospital Universitário Getúlio Vargas, Departamento de Medicina, Manaus, AM, Brasil.; 2 Centro Universitário Para o Desenvolvimento do Alto Vale do Itajaí, Itajaí, SC, Brasil.; 3 Faculdade de Medicina Nova Esperança, João Pessoa, PB, Brasil.; 4 Universidade Federal do Amazonas, Manaus, AM, Brasil.; 5 Universidade Federal de São Paulo, Departamento de Medicina, Disciplina de Medicina de Urgência e Medicina Baseada em Evidências, São Paulo, SP, Brasil.

**Keywords:** Fumarato de Tenofovir Alafenamida, Fumarato de Tenofovir Disoproxil, Doença Renal Crônica, Hepatite B Crônica

## Abstract

**Introdução:**

O tenofovir disoproxil fumarato (TDF) é eficaz no tratamento do vírus da hepatite B (VHB), mas tem sido associado à nefrotoxicidade. Em contraste, o tenofovir alafenamida fumarato (TAF) surgiu como uma alternativa mais segura, reduzindo a exposição renal e mantendo a eficácia antiviral. Esta meta-análise avalia melhorias na função renal após a mudança de TDF para TAF.

**Métodos:**

O estudo foi registrado no PROSPERO (CRD42024565358) e incluiu 10 ensaios clínicos randomizados (ECRs), envolvendo 1.179 pacientes com doença renal crônica (DRC). Foi comparada a função renal antes e após a mudança para TAF.

**Resultados:**

Foram observadas melhorias significativas na taxa de filtração glomerular (TFG), indicando melhora da função renal após a troca. Os achados confirmam que o TAF demonstra um perfil de segurança renal superior em comparação ao TDF, particularmente em tratamentos de longo prazo.

**Conclusão:**

A relevância clínica do TAF para pacientes com VHB e DRC está alinhada com as mudanças recentes nas diretrizes, que têm favorecido seu uso. Apesar de limitações, como a alta heterogeneidade entre os estudos incluídos, esta meta-análise apoia o TAF como uma estratégia de tratamento mais segura para pacientes com VHB e DRC, demonstrando melhores resultados renais e riscos reduzidos de nefrotoxicidade. Esses achados sustentam seu uso mais amplo na prática clínica e destacam a necessidade de mais pesquisas sobre os resultados renais em longo prazo.

## Introdução

O VHB crônico continua sendo um desafio significativo para a saúde global, afetando milhões de pessoas e levando a complicações hepáticas graves, como cirrose e carcinoma hepatocelular^1,2^. A terapia antiviral eficaz a longo prazo é essencial para suprimir a replicação viral e reduzir a progressão da doença^1,3^.

O tratamento atual de primeira linha para o VHB crônico inclui análogos de nucleosídeos/ nucleotídeos — especificamente entecavir, TDF e TAF — bem como o peginterferon^3^. Esses agentes são recomendados por sua eficácia em suprimir a replicação do VHB, reduzir a inflamação hepática e diminuir o risco de cirrose e carcinoma hepatocelular^1,3^. Os análogos de nucleosídeos/ nucleotídeos são particularmente favorecidos devido à sua administração oral e aos perfis de resistência favoráveis^3,4^.

O tenofovir disoproxil fumarato (TDF) tem sido amplamente adotado por sua potente eficácia antiviral^5^. No entanto, seu uso a longo prazo tem levantado preocupações quanto à segurança renal, especialmente em pacientes com doença renal pré-existente ou que necessitam de terapia prolongada^1,4^. O TDF tem sido associado à nefrotoxicidade, incluindo redução da taxa de filtração glomerular (TFG), proteinúria e síndrome de Fanconi. Esses efeitos são atribuídos principalmente ao acúmulo de tenofovir dentro das células tubulares proximais, onde induz disfunção mitocondrial ao esgotar o DNA mitocondrial, interromper a fosforilação oxidativa e prejudicar o metabolismo energético celular. Essa toxicidade mitocondrial pode levar à lesão tubular aguda e à progressão para doença renal crônica, particularmente em pacientes com DRC sublinhada^6,7^.

Para superar as preocupações de segurança renal e óssea associadas ao TDF, o tenofovir alafenamida (TAF) foi desenvolvido como um novo pró-fármaco. Assim como o TDF, o TAF inibe a transcriptase reversa do VHB, mas sua estabilidade aprimorada no plasma e a ativação intracelular seletiva resultam em maior entrega de hepatócitos em doses mais baixas^4,8^. Essa vantagem farmacocinética mantém uma potente atividade antiviral, reduzindo significativamente a exposição sistêmica e minimizando a toxicidade (principalmente toxicidades renais e ósseas)^9^.

Evidências recentes reforçam o perfil de segurança aprimorado do TAF. Um estudo de Sax *et al*. demonstrou que a mudança de TDF para TAF melhora os marcadores de função renal, com reduções na proteinúria, aumentos na taxa de filtração glomerular estimada (TFGe) e níveis mais baixos de biomarcadores de lesão tubular^10^. Essas descobertas são ainda apoiadas por um grande estudo de coorte do mundo real conduzido por Farag et al.^11^, que acompanhou pacientes com hepatite B crônica por até 160 semanas após a mudança para TAF. O estudo mostrou que, em pacientes previamente tratados com TDF, o declínio da TFGe foi interrompido ou mesmo revertido após a mudança para TAF, particularmente naqueles com DRC moderada a avançada (TFGe <60 mL/min). Notavelmente, melhorias também foram observadas em pacientes com DRC estágio 2 (TFGe 60–89 mL/min), sugerindo que os benefícios renais do TAF podem se estender além dos limites atualmente recomendados. Essas descobertas, consistentes em ensaios clínicos e ambientes de mundo real, fortalecem a justificativa para a transição de pacientes em risco para o TAF, a fim de mitigar complicações renais durante a terapia antiviral de longo prazo.

Nesse contexto, a presente meta-análise de braço único teve como objetivo sintetizar dados da última década para avaliar os desfechos renais em pacientes com VHB crônico e DRC que realizaram a mudança de TDF para TAF. Ao focar nessa população de alto risco, o estudo buscou determinar se o TAF fornece uma estratégia antiviral mais segura, porém igualmente eficaz, para o tratamento do VHB a longo prazo.

## Métodos

Esta revisão sistemática com meta-análise foi registrada no *International Prospective Register of Systematic Reviews* (PROSPERO), sob o protocolo CRD42024565358. O estudo foi desenhado seguindo as diretrizes de relato do *Preferred Reporting Items for Systematic Reviews and Meta-analyses* (PRISMA).

### Elegibilidade do Estudo

Foram incluídos estudos que atenderam aos seguintes critérios: (1) ensaios clínicos randomizados (ECRs) ou estudos de coorte que avaliaram a função renal antes e depois da mudança para TAF em pacientes com DRC. Foram excluídos estudos com (1) dados incompletos, (2) transplante de rim e/ou fígado e (3) populações de pacientes sobrepostas. Nenhuma restrição quanto à data de publicação ou ao idioma foi aplicada.

### Estratégia de Busca e Extração de Dados

As bases de dados MEDLINE, *Cochrane* e *Embase* foram pesquisadas sistematicamente em 1º de julho de 2024. A estratégia de busca foi a seguinte: (“hepatite B crônica”) E (“doença renal crônica”) E (“tenofovir alafenamida” OU “TAF”) E (“tenofovir disoproxil fumarato” OU “TDF” OU “Adefovir” OU “ADV”). Foram extraídos dados referentes a: (1) número de pacientes com DRC antes da mudança para TAF e (2) número de pacientes que apresentaram aumento de, pelo menos, um estágio da classificação da DRC, independentemente do aumento absoluto da TFGe, após a mudança de TDF para TAF, durante um período de observação que variou de 24 a 96 semanas. No entanto, os dados individuais de TFGe em nível de paciente não estavam disponíveis nos estudos incluídos, o que impediu uma análise quantitativa das alterações absolutas dessa variável. Todos os artigos identificados foram avaliados sistematicamente com base nos critérios de inclusão e exclusão. A seleção dos estudos e a extração dos dados foram realizadas de forma independente por, pelo menos, dois revisores. E-mails foram enviados aos autores caso os dados não estivessem disponíveis no texto do estudo. As informações sobre o número de pacientes com TFGe aumentada (desfecho principal) foram extraídas por meio da leitura do texto e da disposição em planilhas do Excel^®^. As divergências foram resolvidas por consenso.

### Avaliação da Qualidade

A avaliação da qualidade para esta meta-análise seguiu uma abordagem rigorosa e padronizada para garantir a confiabilidade e a validade dos estudos incluídos. O risco de viés foi avaliado utilizando a ferramenta *Cochrane Risk of Bias 2* (RoB2), aplicada aos ECRs^12^. Além disso, foram realizados um *funnel plot* e o teste de *Egger* ^13^. Cada estudo foi analisado independentemente por dois revisores quanto a possíveis vieses, incluindo viés de seleção, desempenho, detecção e relato. As discrepâncias foram resolvidas por meio de discussão ou pela consulta a um terceiro revisor, quando necessário. Os estudos foram classificados com base na qualidade metodológica, incluindo a adequação da randomização, o cegamento e a completude dos dados dos desfechos.

### Análise de Sensibilidade

Para a análise de sensibilidade, foram realizados diversos testes para avaliar a robustez de nossos achados e determinar se os resultados foram influenciados por estudos específicos ou suposições metodológicas. Primeiramente, foi conduzida uma análise *leave-one-out*, na qual cada estudo foi excluído sequencialmente para avaliar seu impacto no tamanho geral do efeito. Essa abordagem ajudou a identificar se algum estudo exerceu uma influência desproporcional nos resultados da meta-análise. Além disso, foi realizada uma meta-regressão com base nas características basais disponíveis — idade, TFGe e sexo — para explorar fontes potenciais de heterogeneidade. A influência de estudos classificados como de alto risco de viés também foi examinada por meio de uma análise separada, excluindo esses estudos.

### Análise de Dados

Uma abordagem abrangente foi empregada para sintetizar e interpretar os achados dos estudos incluídos. As análises estatísticas foram realizadas usando um modelo de efeitos aleatórios, a fim de considerar a potencial heterogeneidade entre os estudos^13,14^. Foram calculados os efeitos do tratamento para desfechos binários e as diferenças médias (DM) para variáveis contínuas, ambos acompanhados de intervalos de confiança (ICs) de 95%. O teste de *Mantel-Haenszel* foi aplicado a todos os desfechos binários.

Com base nos dados coletados e nos resultados da meta-análise, elaborou-se um gráfico de floresta demonstrando a proporção de cada estudo, expressa como a fração de indivíduos que apresentaram o desfecho de interesse analisado em relação ao número total de participantes de cada estudo.

A heterogeneidade entre os estudos foi avaliada por meio da estatística *I^2^*, com valores de 25%, 50% e 75% interpretados como indicativos de heterogeneidade baixa, moderada e alta, respectivamente. Nos casos de heterogeneidade substancial (*I^2^* > 50%), foram realizadas análises de subgrupos para investigar possíveis fontes de variação, incluindo diferenças nas características dos pacientes, no desenho dos estudos ou na duração do acompanhamento. O viés de publicação foi avaliado por meio da inspeção visual dos gráficos de funil e analisado quantitativamente pelo teste de regressão de *Egger*. A assimetria observada nos gráficos de funil foi considerada indicativa de potencial viés de publicação. Estabeleceu-se a significância estatística em um valor de p bicaudal < 0,05. Todas as análises estatísticas foram realizadas no software R (versão 2024.12.1, build 563), utilizando-se os pacotes meta^15^, metafor^16^ e dmetar^17^ para metanálise, meta-regressão e avaliação de viés, garantindo uma síntese rigorosa e reprodutível dos dados.

## Resultados

Nossa busca sistemática identificou inicialmente 69 artigos potenciais, conforme ilustrado na Figura 1. Após a remoção de duplicatas e a exclusão de estudos que não atendiam aos critérios de inclusão com base na triagem de títulos e resumos, 13 artigos permaneceram para leitura na íntegra. Destes, 10 ECRs preencheram todos os critérios de elegibilidade e foram incluídos na meta-análise. Um estudo foi excluído por se tratar de um protocolo, e outros dois não possuíam dados de desfecho suficientes. No total, a meta-análise incluiu dados de 1.179 pacientes com DRC que realizaram a mudança de TDF para TAF.

**Figura 1.** Fluxograma PRISMA de seleção dos estudos.

O risco de viés foi avaliado por meio da ferramenta RoB 2 (Figura 2). O viés de publicação foi examinado utilizando o gráfico de funil Figura 3) e o teste de *Egger*, que não indicou assimetria significativa (intercepto = -1,38; IC 95%: -4,40 a 1,63; p = 0,39), sugerindo baixa probabilidade de viés de publicação.

**Figura 2.** Risco de viés *Cochrane* (RoB2).

**Figura 3.** Gráfico de funil.

A meta-análise concentrou-se em avaliar o impacto da mudança de TDF para TAF na função renal de pacientes com VHB e DRC. Embora o TDF seja eficaz na supressão do VHB, seu uso prolongado tem sido associado à nefrotoxicidade, incluindo reduções na TFG e outras complicações renais. O TAF, um pró- fármaco mais recente do tenofovir, fornece eficácia antiviral equivalente com exposição sistêmica significativamente menor, potencialmente oferecendo melhores resultados renais.

A análise combinada demonstrou uma melhora significativa na TFG após a mudança para TAF. Entre os 1.179 pacientes incluídos, o *odds ratio* (OR) para melhora da TFG foi de 24,13 (IC 95%: 18,31–31,09; p < 0,01). A proporção de contribuição de cada estudo para o resultado foi semelhante, indicando um benefício estatisticamente significativo. Apesar da alta heterogeneidade (*I^2^* = 79%), a direção e a magnitude do efeito foram consistentes entre os estudos, corroborando o perfil de segurança renal do TAF em comparação ao TDF (Figura 4). Os dados detalhados usados na análise são fornecidos no material suplementar.

**Figura 4:** Gráfico de floresta.

É importante observar que nenhum desfecho secundário — como progressão para doença renal terminal (DRT), marcadores de lesão tubular ou indicadores de saúde óssea — foi relatado de forma consistente nos estudos. Como tal, esta meta-análise limitou-se a avaliar as alterações na TFG.

Uma alta heterogeneidade foi observada em quatro dos estudos incluídos — Hosaka et al.^18,19^, Farag et al.^11^, e Ogawa et al.^20^ — o que pode ser atribuído a vários fatores. Entre eles está o fato de que a TFGe não foi o desfecho primário em alguns estudos, o tamanho reduzido das amostras, a ampla variabilidade etária, as origens étnicas diversas, a presença de comorbidades além da DRC e a exposição prévia a terapias antivirais combinadas antes da transição de TDF para TAF. Apesar dessas fontes de variabilidade, as características basais das populações estudadas permaneceram amplamente alinhadas aos nossos critérios de inclusão, apoiando a consistência interna da análise. De acordo com a estrutura GRADE, esse alinhamento reforça a robustez geral dos achados.

Para explorar ainda mais o impacto da heterogeneidade, foram realizadas análises de sensibilidade. A exclusão do estudo mais heterogêneo produziu um OR para melhora da TFGe de 22,29 (IC 95%: 17,75–27,61; p < 0,01), mantendo a significância estatística (Figura 5). Quando todos os quatro estudos altamente heterogêneos foram excluídos, o OR foi ajustado para 18,70 (IC 95%: 14,77–23,40; p = 0,16), indicando precisão reduzida, mas ainda sugerindo uma tendência favorável ao benefício renal com o uso de TAF.

**Figura 5.** Gráfico de floresta sem o estudo mais heterogêneo.

Além disso, foi conduzida uma meta-regressão de efeitos mistos para investigar se as variáveis basais poderiam explicar parte da heterogeneidade observada. Foram encontradas associações significativas com a TFGe basal (τ^2^ = 0,15; τ = 0,39; intercepto = -1,35; p < 0,001) e com a idade (τ^2^ = 0,07; τ = 0,28; intercepto = -1,80; p < 0,001), enquanto nenhuma influência significativa foi detectada para o sexo masculino (p = 0,80) ou feminino (p = 0,77). Esses resultados sugerem que variáveis adicionais não medidas podem ter contribuído para a variabilidade entre os estudos.

Além da significância estatística, os achados ressaltam a relevância clínica da mudança de TDF para TAF em pacientes com VHB e DRC. Dado o risco de nefrotoxicidade estabelecido e associado ao uso prolongado de TDF, a transição para TAF não apenas melhora os marcadores da função renal, como a TFGe, mas também pode reduzir o dano renal a longo prazo. Esses resultados fortalecem as preferências clínicas atuais que favorecem o uso de TAF, particularmente em pacientes com insuficiência renal pré-existente ou em risco de disfunção renal devido à terapia antiviral de longo prazo. No geral, as evidências dão suporte à priorização do TAF como opção terapêutica preferida no tratamento do VHB em pacientes com função renal comprometida.

## Discussão

Esta revisão sistemática e meta-análise avaliou os desfechos renais em 1.179 pacientes com DRC e infecção crônica pelo VHB que realizaram a transição de TDF para TAF em 10 ensaios clínicos randomizados. O achado primário foi uma melhora significativa na TFGe após a troca, com uma proporção substancial de pacientes experimentando uma melhora de pelo menos um estágio da DRC. Esses resultados fornecem suporte robusto para a segurança renal do TAF e reforçam as preferências clínicas atuais para seu uso no tratamento do VHB em pacientes com insuficiência renal.

Embora o TDF seja eficaz na supressão viral, seu uso prolongado tem sido consistentemente associado à nefrotoxicidade. Em contraste, o TAF fornece o metabólito ativo de forma mais eficiente e em concentrações sistêmicas mais baixas, reduzindo a exposição renal e a toxicidade associada. Nossos achados reforçam esse raciocínio farmacológico, apoiando a preferência pelo TAF em pacientes com disfunção renal existente ou que necessitam de terapia antiviral prolongada^1,2^.

No Brasil, de acordo com o *Protocolo Clínico e Diretrizes Terapêuticas de Hepatite B e Coinfecções* de 2023, o uso de TAF é recomendado para o tratamento do VHB em usuários prévios de lamivudina nas seguintes condições: (1) cirróticos com escore de *Child-Pug* A; (2) presença de microalbuminúria, proteinúria persistente, fosfato sérico <2,5 mg/dL, TFG <60 mL/min, redução da TFG >25% após o início do tratamento, uso de terapia imunossupressora ou quimioterapia nefrotóxica; (3) osteoporose, fratura óssea patológica, usuários crônicos de corticosteroides e alto escore FRAX; (4) pacientes transplantados ou em terapia imunossupressora^21^.

É importante ressaltar que as análises de sensibilidade e de meta-regressão demonstraram que os benefícios renais observados com o TAF permaneceram consistentes, apesar da alta heterogeneidade entre os estudos. Essa heterogeneidade, observada em trabalhos como os de Hosaka et al.^18,19^, Fung et al.^11^ e Ogawa et al.^20^, foi atribuída a diferenças no desenho dos estudos, características basais, comorbidades e regimes de tratamento prévios. No entanto, a consistência metodológica e os perfis de pacientes comparáveis entre os estudos nos permitiram manter a confiança nas estimativas de efeito combinado. Mesmo quando os estudos mais heterogêneos foram excluídos, o benefício renal do TAF persistiu, destacando a robustez dos achados.

Apesar da força das evidências, esta meta-análise também revelou limitações importantes na literatura atual. Notadamente, os resultados secundários — como progressão para DRT, marcadores de disfunção tubular ou parâmetros de saúde óssea, particularmente relevantes devido ao impacto conhecido dos análogos de nucleotídeos na densidade mineral óssea — foram relatados de forma inconsistente ou estavam totalmente ausentes. Essa lacuna limita uma avaliação mais abrangente do perfil de segurança completo do TAF e ressalta a necessidade de que estudos futuros incorporem desfechos renais e esqueléticos mais amplos.

É importante ressaltar que a Organização Mundial da Saúde (OMS) reafirmou a meta global de eliminar a hepatite B como ameaça à saúde pública até 2030^22^. No entanto, o progresso tem sido lento, com menos de 3% dos cerca de 254 milhões de pessoas que vivem com VHB crônico atualmente recebendo tratamento^1,2,22^. Nesse contexto, estratégias que priorizem não apenas a eficácia antiviral, mas também a segurança e a tolerabilidade em longo prazo, como a ampliação do acesso ao TAF, são essenciais^1^.

As diretrizes atualizadas da OMS para 2024 enfatizam a simplificação dos algoritmos terapêuticos e a expansão da elegibilidade para o tratamento, especialmente em países de baixa e média renda, onde diagnósticos complexos e o monitoramento de longo prazo geralmente não são viáveis. A integração de considerações sobre segurança renal nessas estruturas simplificadas pode ajudar a otimizar os resultados terapêuticos e prevenir complicações evitáveis^22^. Os formuladores de políticas devem considerar a inclusão de resultados renais em análises de custo-efetividade e nos protocolos nacionais de tratamento, de modo a apoiar um acesso mais amplo ao TAF e, assim, contribuir para a agenda de eliminação até 2030.

Este estudo também se alinha aos esforços da comunidade científica em priorizar a segurança dos medicamentos, juntamente com sua eficácia. Dada a nefrotoxicidade bem documentada associada ao uso prolongado do TDF^9^, esta meta-análise consolida as evidências do perfil de segurança renal superior do TAF, reforçando sua inclusão nas diretrizes clínicas para o tratamento da hepatite B. Esses achados não apenas validam a transição contínua para o TAF na prática clínica, mas também apoiam seu uso mais amplo entre pacientes com insuficiência renal pré-existente. Além disso, esta meta-análise fortalece a base de evidências ao sintetizar informações provenientes de diversos estudos, abordando lacunas existentes nos dados do mundo real e auxiliando os profissionais de saúde na tomada de decisões terapêuticas baseadas em evidências. Ao contribuir para o crescente corpo de pesquisas voltadas à redução da toxicidade de órgãos induzida por medicamentos, essas descobertas também podem impulsionar mais inovações em terapias antivirais e em estratégias de saúde pública.

No tratamento do HIV-1, os esquemas contendo TAF já demonstraram não inferioridade na eficácia antiviral, oferecendo maior segurança em relação aos resultados do metabolismo renal, ósseo e lipídico. Em uma meta-análise conduzida por Tao et al.^23^, um OR de 0,31 (IC 95%: 0,18–0,55) indicou um risco significativamente menor de eventos renais ao comparar o TAF com o TDF. Por outro lado, outra meta-análise publicada no mesmo ano por Pilkinton et al.^24^ não encontrou diferença significativa nas taxas de eventos adversos renais entre os grupos que receberam regimes de HIV contendo TAF ou TDF. No entanto, nesse estudo, o número de eventos renais relatados foi relativamente pequeno (apenas 3 de 4.665 pacientes no grupo TAF e 6 de 4.227 no grupo TDF), potencialmente limitando suas conclusões.

Este estudo apresenta diversas limitações. Mais notavelmente, a ausência de um grupo de controle que compare diretamente o uso contínuo de TDF com a mudança para TAF, o que limita a capacidade de estabelecer conclusões comparativas definitivas. Além disso, a análise incluiu apenas pacientes com DRC e infecção crônica por VHB, o que pode restringir a generalização dos achados para populações mais amplas de VHB e contribuir para a heterogeneidade observada. A elevada heterogeneidade entre os estudos provavelmente reflete a variabilidade nas características basais dos pacientes, desenhos de estudo, comorbidades e durações de acompanhamento. Ademais, nem todos os estudos forneceram dados completos sobre as alterações na TFGe, o que pode ter influenciado a precisão das estimativas agrupadas.

Apesar dessas limitações, os achados fornecem informações importantes sobre o perfil de segurança renal do TAF e ressaltam seu potencial como uma alternativa mais segura ao TDF, particularmente para pacientes com função renal comprometida. A consistência das melhorias observadas na TFGe sustenta a relevância clínica da transição para o TAF nessa população vulnerável.

Além disso, o presente estudo não avaliou outros desfechos clinicamente significativos relacionados à DRC e à nefrotoxicidade. Embora a TFGe seja um marcador central da função renal, ela não abrange todo o espectro da lesão renal. Desfechos relevantes, como progressão para DRT, presença de tubulopatia, fosfatúria, alterações no metabolismo ósseo e distúrbios do fósforo sérico, não foram relatados de forma consistente nos estudos incluídos e, portanto, não puderam ser analisados. Futuros ECRs, com medidas de resultados mais amplas, inclusão de grupos de controle e populações de pacientes mais diversificadas, são necessários para fornecer uma avaliação mais abrangente da segurança renal e metabólica da terapia antiviral de longo prazo — particularmente no contexto da toxicidade tubular associada ao tenofovir.

## Conclusão

Este estudo fornece evidências robustas que apoiam a vantagem de segurança renal do TAF em relação ao TDF em pacientes com DRC e hepatite B crônica. Embora limitado pela ausência de um grupo de controle direto e pela falta de dados sobre desfechos renais adicionais — como progressão para DRT e marcadores de disfunção tubular —, a melhora consistente na TFGe em todos os estudos sugere que o TAF é uma alternativa mais segura para pacientes com risco aumentado de nefrotoxicidade.

Esses achados ressaltam o valor clínico da incorporação do TAF nas estratégias de tratamento voltadas a populações vulneráveis e apoiam sua adoção mais ampla nas diretrizes de manejo do VHB, particularmente em ambientes nos quais a preservação renal a longo prazo é crítica. Pesquisas futuras devem ter como objetivo incluir populações de pacientes mais diversificadas e avaliar uma gama maior de desfechos renais e metabólicos para validar e expandir ainda mais esses resultados. Essas evidências serão essenciais para informar estratégias antivirais baseadas em precisão e conscientes da função renal, num esforço contínuo para otimizar o tratamento do VHB.

## Data Availability

Os dados que sustentam os achados deste estudo estão disponíveis nas informações de apoio deste artigo.

## Material Suplementar

O seguinte material online está disponível para o presente artigo:

Dados dos estudos incluídos na meta-análise.
